# Giant Filiform Polyposis, A Rare Entity in a Patient of Ulcerative Colitis: A Case Report

**DOI:** 10.7759/cureus.6125

**Published:** 2019-11-11

**Authors:** Zafar Ali, Salma Iltaf

**Affiliations:** 1 Histopathology, Shifa International Hospital, Islamabad, PAK

**Keywords:** filiform polyposis, inflammatory bowel disease, ulcerative colitis

## Abstract

Filiform polyposis (FP) of the colon is a distinct and uncommon entity that is occasionally encountered in patients with a history or evidence of inflammatory bowel disease. It is morphologically characterized by multiple, slender, worm-like projections consisting of submucosal cores lined with normal mucosa. Here, we report a case of FP in a 43-year-old middle-aged man with a history of inflammatory bowel disease (ulcerative colitis).

## Introduction

Filiform polyposis (FP) is most frequently secondary to a post-inflammatory reparative process and may occur in 10%-20% of the cases of inflammatory bowel disease in which chronic inflammation of the large bowel mucosa with repeated ulceration and healing may lead to the formation of worm-like polypoid projections [1-3].

The term was first coined by Appleman et al. who used it to describe a syndrome involving the radiological appearance of numerous, long, slender, worm-like, or filiform defects in the colon [4].

FP typically presents as one to hundreds of uniform, slender, arborizing, vermiform projections of the bowel mucosa and submucosa lined by normal or inflamed mucosa. These polyps are thin, straight-shaped, resembling the stalk of polyps without the heads. In rare cases, polyps coalesce and a large tumor mass, which may measure over 15 mm, known as giant filiform polyposis (GFP), is found.

## Case presentation

**Figure 1:**
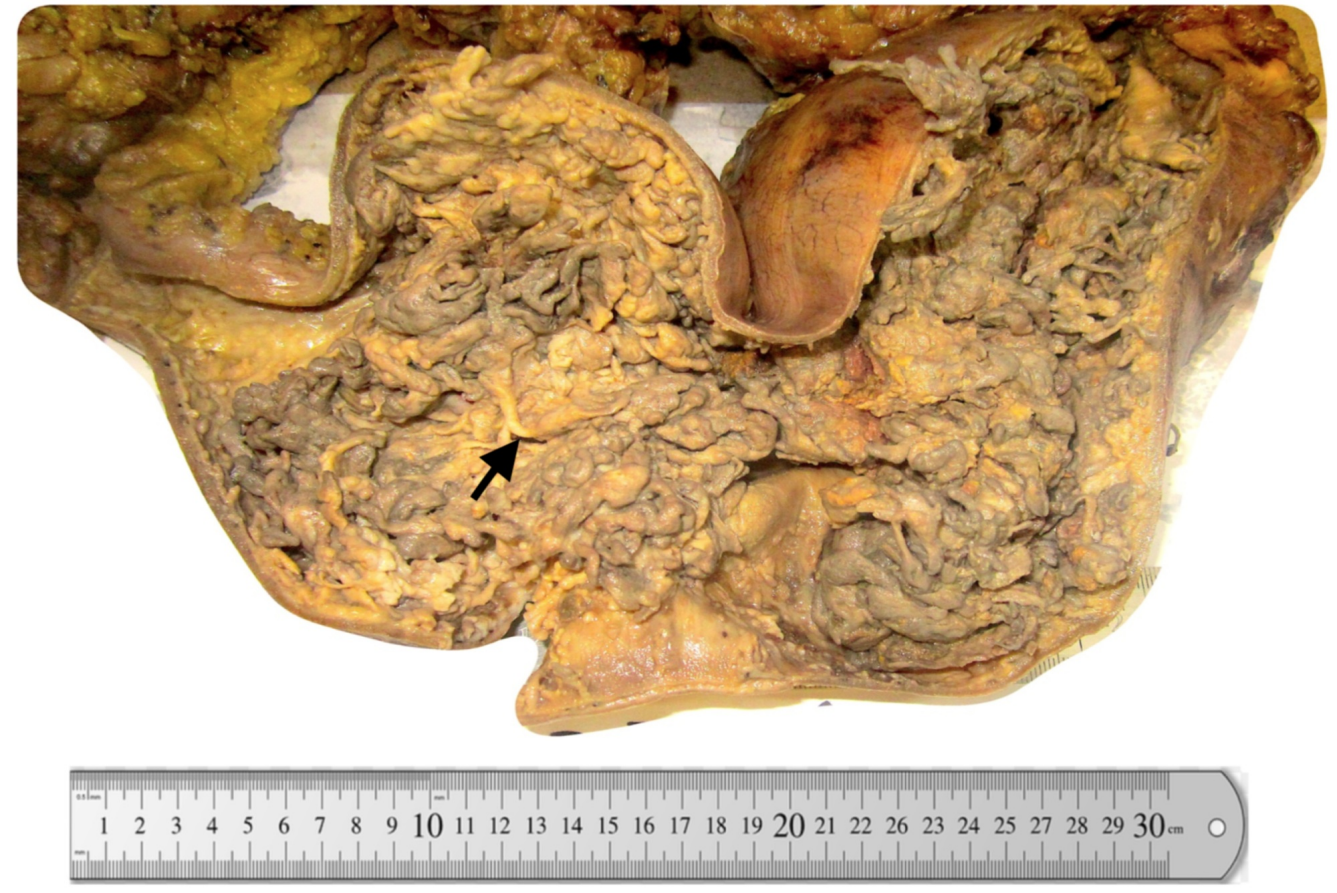
Numerous polyps, more than 100, up to 2.0 cm in size, involving the entire colon

The patient presented with per rectum bleeding, no family history of colon polyps, colon cancer, or inflammatory bowel disease (IBD). He received steroid therapy for an unknown period on a clinical basis and remained symptomatic. Later, multiple colonic biopsies were done and revealed chronic active colitis with the possibility of IBD, most likely ulcerative colitis. The patient underwent total colectomy. The specimen showed numerous polyps, more than 100, up to 2.0 cm in size (Figure 1).

**Figure 2:**
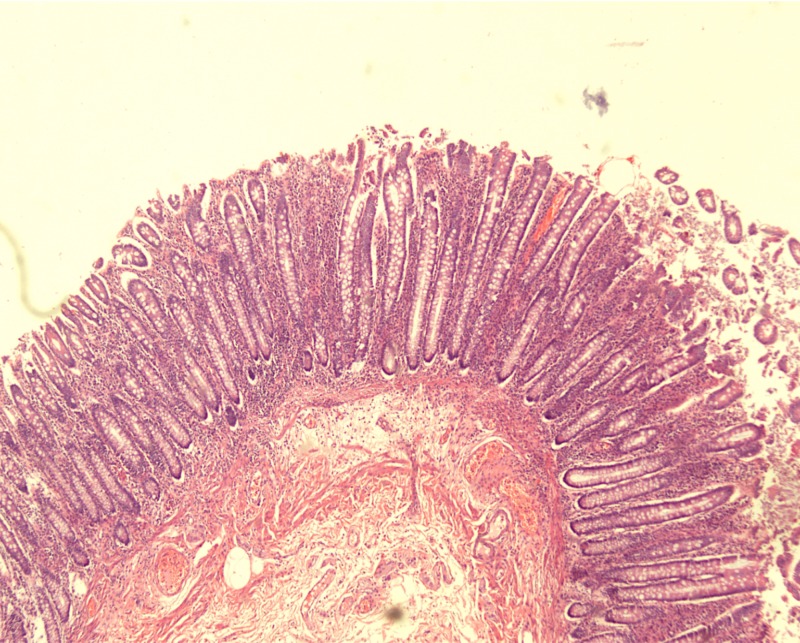
Low-power view showing slender, worm-like projections consisting of submucosal cores lined with normal mucosa (x40 magnification)

**Figure 3:**
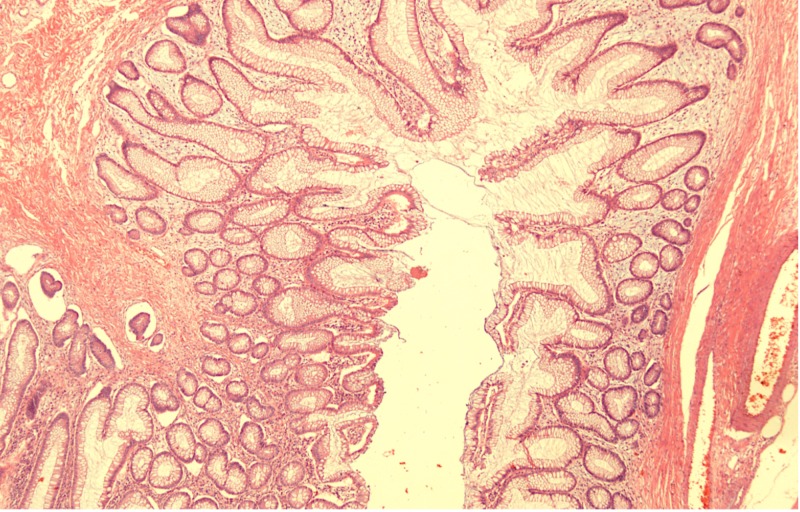
Low-power view of sampled polyps with elongated projections of colonic mucosa, showing branched glands and cystic crypts (x40 magnification)

Histological examination of the sampled polyps showed elongated projections of colonic mucosa showing branched elongated glands and cystic crypts. The central core was composed of submucosal tissue containing vessels, nerves, and lymphoid aggregates (Figures 2-3).

There were areas of ulceration and abscess formation. The surrounding mucosa shows crypt abscesses, cryptitis, and basal plasmacytosis. There are areas of ulceration and abscess formation. At places, the infiltrate is invading the muscle layer. No dysplasia or malignancy was observed in the mucosa covering the polyps or surrounding them.

## Discussion

We report a case of previously undiscovered polyposis in the entire colon, with a definite history of IBD, i.e ulcerative colitis. This report is intended to characterize the clinical and pathologic features of filiform polyposis. Filiform polyposis (FP) is most frequently secondary to a post-inflammatory reparative process of the large bowel mucosa [5]. FP is rare in the pediatric population with few cases reported [6].

FP typically presents as one to hundreds of uniform, slender, arborizing, vermiform projections of the bowel mucosa and submucosa lined by normal or inflamed mucosa. These polyps are thin, straight-shaped, resembling the stalk of polyps without the heads. When these polyps form tumor-like masses, as in our case, they are called giant filiform polyposis (GFP). Grossly, these giant polyps may be mistaken for malignancy. Microscopically, these polypoidal structures are lined by bland-appearing epithelium with no evidence of dysplasia. The adjacent mucosa will show changes of chronic active inflammation as a sequela of IBD. The gross identification and knowledge of this entity is essential for making a correct diagnosis.

Although the presence of dysplasia or malignant transformation has never been reported in FP, in some cases, the polyps are difficult to distinguish from villous adenomas, and biopsies are needed to make the diagnosis [7]. Our case did not show any evidence of dysplasia or invasive malignancy. Partial colectomies should be reserved for complicated cases of symptomatic inflammatory polyps or when malignancy cannot be excluded [8]. For example, if it is thought that FP has developed in association with active IBD, pre-emptive surgical resection would seem reasonable as in our case, particularly given the increased risk of colon cancer with ulcerative colitis and Crohn’s disease [9-11]. However, in asymptomatic patients without a history who are found to have FP, observation is the best option. When patients with inflammatory polyposis require surgical management, it is important to evaluate the margins of resection because inflammatory polyposis can recur in the presence of acute inflammation or residual disease at the resected margins.

## Conclusions

Giant filiform polyposis can grossly mimic adenomatous polyps or colorectal carcinoma. It should be considered in the differential diagnosis of patients presenting with polyposis in IBD. A thorough sampling of the specimen is required to exclude dysplasia or invasive malignancy. A periodic, close follow-up is suggested.
